# Different Stages of Quiescence, Senescence, and Cell Stress Identified by Molecular Algorithm Based on the Expression of Ki67, RPS6, and Beta-Galactosidase Activity

**DOI:** 10.3390/ijms22063102

**Published:** 2021-03-18

**Authors:** Nicola Alessio, Domenico Aprile, Salvatore Cappabianca, Gianfranco Peluso, Giovanni Di Bernardo, Umberto Galderisi

**Affiliations:** 1 Department of Experimental Medicine, Luigi Vanvitelli Campania University, 80138 Naples, Italy; nicola.alessio@unicampania.it (N.A.); domenico.aprile@unicampania.it (D.A.); gianni.dibernardo@unicampania.it (G.D.B.); 2 Department of Precision Medicine, Luigi Vanvitelli Campania University, 80138 Naples, Italy; salvatore.cappabianca@unicampania.it; 3 Research Institute on Ecosystems (IRET), CNR, 80131 Naples, Italy; gianfranco.peluso@cnr.it; 4 Sbarro Institute for Cancer Research and Molecular Medicine, Center for Biotechnology, Temple University, Philadelphia, PA 19122, USA; 5 Genome and Stem Cell Center (GENKOK), Erciyes University, Kayseri 38280, Turkey

**Keywords:** senescence, quiescence, cell cycle, mesenchymal stem cells

## Abstract

During their life span, cells have two possible states: a non-cycling, quiescent state (G0) and a cycling, activated state. Cells may enter a reversible G0 state of quiescence or, alternatively, they may undergo an irreversible G0 state. The latter may be a physiological differentiation or, following a stress event, a senescent status. Discrimination among the several G0 states represents a significant investigation, since quiescence, differentiation, and senescence are progressive phenomena with intermediate transitional stages. We used the expression of Ki67, RPS6, and beta-galactosidase to identify healthy cells that progressively enter and leave quiescence through G0-entry, G0 and G0-alert states. We then evaluated how cells may enter senescence following a genotoxic stressful event. We identified an initial stress stage with the expression of beta-galactosidase and Ki67 proliferation marker. Cells may recover from stress events or become senescent passing through early and late senescence states. Discrimination between quiescence and senescence was based on the expression of RPS6, a marker of active protein synthesis that is present in senescent cells but absent in quiescent cells. Even taking into account that fixed G0 states do not exist, our molecular algorithm may represent a method for identifying turning points of G0 transitional states that continuously change.

## 1. Introduction

Within tissues and organs, some cells are cycling, while others are in a non-proliferative state. This non-proliferative phase (G0) may be a temporary condition (quiescence). Alternatively, cells may definitively arrest proliferation and undergo a differentiation process that will not allow re-entering the cell cycle. These conditions are responses to several anti-mitogenic cues, which include extrinsic environmental signals and intrinsic regulatory mechanisms [1,2].

Some extrinsic and intrinsic signals may induce cell stress by altering physiological cellular conditions with metabolic alteration and damage to macromolecules (DNA, RNA, proteins, lipids). Cells attempt to cope with such stress and restore functional integrity; however, in some cases, the stress leads to permanent cell-machinery injury, and cells become senescent [3,4]. Senescent cells definitively leave the cell cycle, lose their original functions, and acquire peculiar new activities to counteract cancer growth and contribute to tissue repair and development. Beyond these tasks, senescent cells have a dark side, since they contribute to aging and, in some settings, promote cancer progression rather than blocking it [3,4].

Discrimination between the several G0 states is of paramount importance for evaluating whether a given cell is in quiescence rather than in senescence. Furthermore, it is of interest to ascertain whether a stressful event leads to permanent or temporary cell injury. Several markers have been proposed as indicators of quiescence, senescence, and stress status. The expression of Ki67 and the ribosomal protein S6 (RPS6), along with the evaluation of beta-galactosidase activity, are the most widely used “tools” to identify quiescent, senescent, and stressed cells [5,6,7,8].

The identification of G0 states represents a significant investigation since quiescence, differentiation, and senescence are progressive phenomena with intermediate stages, and the above-indicated markers have been suggested to have some bias. The Ki67 protein contributes to normal cell-cycle progression and is considered a marker of cycling cells and not of resting cells [8,9]. Nevertheless, there are findings showing that Ki67 may be expressed at low levels in quiescent cells [10].

One of the most energy-consuming processes is protein synthesis; quiescent cells, having low energy storage, adjust energy demands by reducing protein production. In contrast, senescent cells perform active protein synthesis to accomplish their several tasks [5,7,11]. Upon activation of protein synthesis by extracellular or intrinsic stimuli, RPS6 is phosphorylated by S6 kinases that belong to the mTOR signaling pathway [12,13]. Phosphorylated RPS6 (pRPS6) is considered an indicator of active protein synthesis and has been proposed as a marker that allows discrimination between senescent and quiescent cells [5].

The most widely used senescence marker is the senescence-associated beta-galactosidase (SA-β-gal), a eukaryotic hydrolase localized in the lysosomes. The activity of SA-β-gal is measured at pH 6.0, while, physiologically, beta-galactosidase is active at pH 4.0, the typical lysosomal acid environment [6]. SA-β-gal activity is associated with a senescent phenotype, but is not a causative circumstance since its silencing does not reverse senescence [14]. This observation may represent a limit for the use of SA-β-gal as a marker of senescence; moreover, there are reports showing that this enzyme is also highly active following persistent stress, such as forced crowding culture conditions, temperature, and pH switch-over [15].

In this context, the above reported findings evidence that: i) the Ki67 marker may not unequivocally identify cycling cells; and ii) the SA-β-gal activity is not a senescence-specific marker.

A clear discrimination among cycling, quiescent and senescent cells may require the contemporary evaluation of both markers along with determination of protein synthesis status through assessment of RPS6 phosphorylation. We provide evidence that the combined analysis of Ki67 and pRPS6 expression, along with the evaluation of beta-galactosidase activity, may be useful for identifying cells entering and leaving the cell cycle, as well as senescent cells.

## 2. Results

### 2.1. Development of a Working Model to Evaluate Cell-Cycle Exit and Cell-Cycle Re-Entry

We performed our experiments on proliferating human mesenchymal stromal cells (MSCs). MSCs contain a subpopulation of stem cells, which are differentiated as adipocytes, chondrocytes, and osteocytes. MSCs secrete numerous cytokines and growth factors, thus contributing to the homeostatic maintenance of many organs [16]. The study of quiescence and senescence of MSCs is very important, given their key role in the body’s homeostasis.

**Figure 1:**
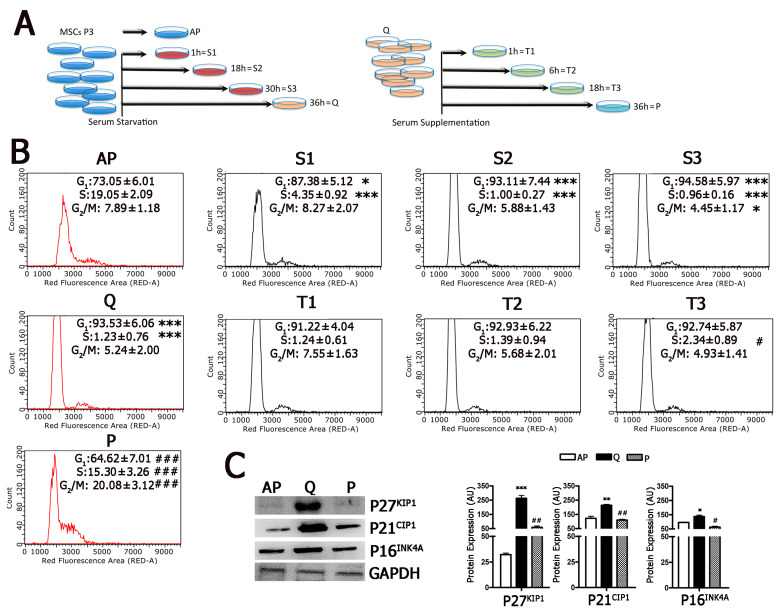
Follow-up of cell-cycle exit and cell-cycle re-entry. Panel (**A**): Graphic summary of the experimental procedure. MSCs at passage 3 (P3) were serum starved for 36 h and, at different time points (AP, S1, S2, S3, Q), cell samples were collected for analysis. Cells were then incubated in a medium containing serum and were cultivated for another 36 h. At different time points (T1, T2, T3, P), samples were collected for evaluation. Panel (**B**): Cell-cycle profiles of MSC samples collected at different time points during starvation and serum re-supplementation. The * indicates the statistical difference between AP, chosen as reference, and S1, S2, S3, and Q (* *p* < 0.05, ** *p* < 0.01, *** *p* < 0.001). The # indicates the statistical difference between Q, chosen as the reference, and T1, T2, T3, and P (# *p* < 0.05, ## *p* < 0.01, ### *p* < 0.001). Panel (**C**): Western blots of MSC samples collected at different time points during starvation and serum re-supplementation. The graphs show the quantification of Western blot bands performed by using GAPDH as the loading control. The * indicates the statistical difference between AP, chosen as the reference, and Q (* *p* < 0.05, ** *p* < 0.01, *** *p* < 0.001). The # indicates the statistical difference between Q, chosen as the reference, and P (# *p* < 0.05, ## *p* < 0.01).

The experimental plan for our study is depicted in Figure 1A and Figure 3A. Asynchronous proliferating MSCs (70% confluence) were serum starved for 36 h to reach quiescence; then, we supplemented serum to allow cell-cycle re-entry. We collected cell samples at several time points during serum starvation and following serum re-supplementation. Proliferating MSCs showed 19.0% of cells in the S-phase, as detected by cell-cycle analysis with flow cytometry (Figure 1B). This percentage abruptly decreased to 4.3% after 1 h of serum starvation (time S1) and become negligible 36 h post-starvation (time Q). At the same time, the G1/G0 peak passed from 73.0–93.5% (Figure 1B).

Serum supplementation produced a re-entry of cells into the cycling state with a slow kinetics; the doubling of S-phase cells occurred 18 h post-supplementation (time T3), and then this percentage grew exponentially to 15.3% at 36 h following serum addition (time P) (Figure 1B). It should be underlined that, at time P, when cells are actively proliferating, the cell-cycle profile did not overlap that of asynchronously proliferating MSCs (time AP). This result can be explained considering that the two cell-cycle patterns referred to asynchronously and synchronously growing cells.

We evaluated the expression of cell-cycle regulators in asynchronously cycling cells (time AP), in cells at time Q and at time P. We focused our attention on P16^INK4A^, P27^KIP1^, and P21^CIP1^ since several findings have indicated that these three cyclin kinase inhibitors (CKIs) play a role in MSC quiescence [17,18,19]. At time Q, cells showed significant changes in the expression of CKIs compared to cycling cells and cells that re-entered the cell cycle (time P) (Figure 1C). This result confirmed that serum starvation induced cell-cycle exit and its re-supplementation produced cell-cycle entry of MSCs.

### 2.2. Cells Enter and Leave Quiescence through Intermediate Stages

**Figure 2:**
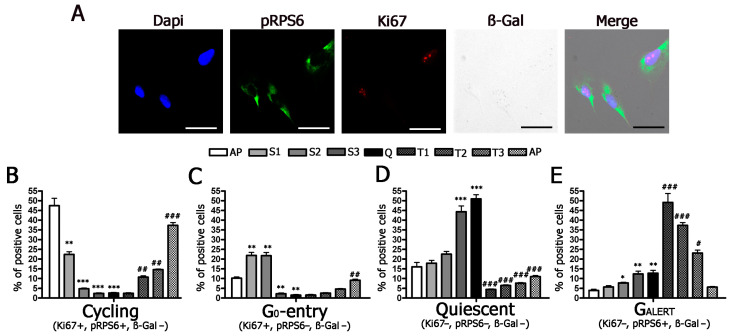
Molecular characterization of cycling and quiescent cells. Panel (**A**): Representative micrographs of MSCs stained to identify nuclei (DAPI), pRPS6, and Ki67 and to evaluate SA-β-gal activity. See also Supplemental File S1. The scale bar corresponds to 100 microns. Panels (**B**–**E**): Graphs show the percentage of cycling, G0-entry, quiescent and G0-alert cells at different time points (AP, S1, S2, S3, Q, T1, T2, T3, P). Below each graph, the molecular algorithm we used to identify the different phenotypes is indicated. In each graph, the * indicates the statistical difference between AP, chosen as the reference, and S1, S2, S3, and Q (* *p* < 0.05, ** *p* < 0.01, *** *p* < 0.001). The # indicates the statistical difference between Q, chosen as the reference, and T1, T2, T3, and P (# *p* < 0.05, ## *p* < 0.01, ### *p* < 0.001).

Having demonstrated that our experimental conditions may represent a working model for evaluating cell-cycle exit and cell-cycle re-entry, we analyzed the percentage of cells expressing Ki67 and pRPS6 at different time points. We focused our attention on healthy non-stressed cells that did not express SA-β-gal and identified four different cell populations: Ki67 (+) pRP6 (+), Ki67 (+) pRPS6 (−), Ki67 (−) pRPS6 (−), and Ki67 (−) pRP6 (+) (Table 1 and Figure 2). The Ki67 (+) pRPS6 (+) cells represent cycling cells that have active protein synthesis; the Ki67 (−) pRPS6 (−) cells are quiescent (G0) cells that have left the cell cycle and have limited protein synthesis. We hypothesized that Ki67 (+) pRP6 (−) cells are those entering quiescence (G0-entry), while Ki67 (−) pRP6 (+) cells correspond to those leaving quiescence (G0-alert).

The asynchronously proliferating MSCs showed 42.0% of cycling cells, and this percentage progressively declined to very low levels at time Q (2.7%) (Figure 2A,B). Then, following serum supplementation, this percentage slowly increased to reach 36.9% (Figure 2B). The quiescent (G0) cells accounted for 16.0% of asynchronously proliferating cells and increased to reach 47.9% at time Q, then declined immediately following serum supplementation (time T1) and remained low in the presence of serum (Figure 2D). It is particularly interesting that the G0-entry cells also increased following serum starvation; this increase occurred at earlier time points (S1 and S2) compared to G0 cells (time S3 and S4) (Figure 2C). These results are in line with the hypothesis that cells enter into a definitive G0 state through progressive preliminary stages. Serum starvation induced a global increase in cells in different G0 stages; indeed, we also noticed an increase in G0-alert cells up to 13.3% at time Q (Figure 2E). One hour after serum addition (time T1), we detected a highly significant increase in G0-alert cells (52.4%); this value declined later along with the increase in the percentage of cycling cells (Figure 2E). Globally, these results evidenced that serum starvation initially induced an increase in G0-entry cells, followed by augmentation of cells in a true G0 state. This event was associated with a progressive reduction in the percentage of cycling cells. The cell-cycle re-entry showed a preliminary peak of G0-alert cells that gradually declined as the percentage of cycling cells increased.

It should be underlined that, at every time point, the sum of G0, G0-entry, and G0-alert cells, which we identified by Ki67 and pRPS6 immunostaining, was lower than the G1/G0 peaks we found by flow cytometry, since these peaks contained both the G0 cells and those that were in the G1 Phase.

### 2.3. Cells Enter Senescence through Intermediate Stages

**Figure 3:**
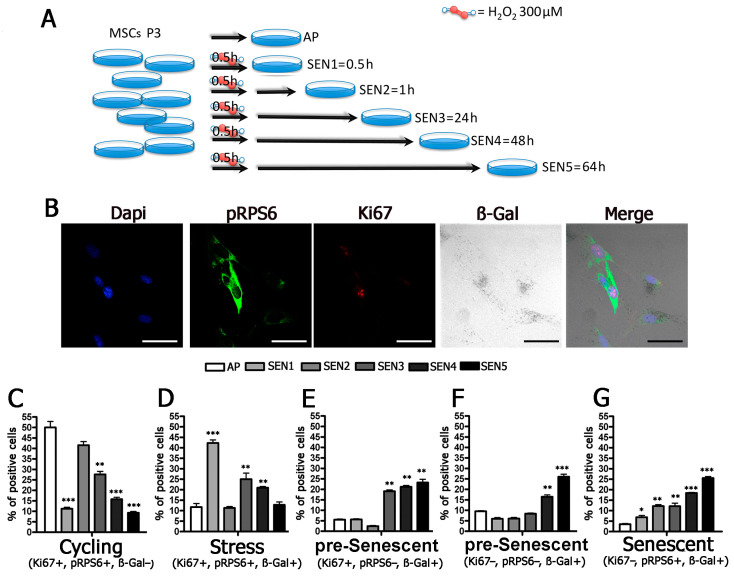
Molecular characterization of stressed and senescent cells. Panel (**A**): Graphic summary of experimental procedure. MSCs at passage 3 (P3) were treated for 0.5 h with H_2_O_2_ and then incubated in cell culture medium for 64 h. At different time points (AP, SEN1, SEN2, SEN3, SEN4, SEN5), cell samples were collected for analysis. Panel (**B**): Representative micrographs of MSCs stained to identify nuclei (DAPI), pRPS6, and Ki67 and to evaluate SA-β-gal activity. The scale bar correspond to 100 microns. See also Supplemental File S3. Panels (**C**–**G**): Graphs show the percentage of cycling, stressed, pre-senescent and senescent cells at different time points (AP, SEN1, SEN2, SEN3, SEN4, SEN5). Below each graph, the molecular algorithm we used to identify the different phenotypes is indicated. In each graph, the * indicates the statistical difference between AP, chosen as the reference, and the other time points (* *p* < 0.05, ** *p* < 0.01, *** *p* < 0.001).

Previously, we demonstrated that MSCs become senescent following H_2_0_2_ treatment [20]. We then evaluated the several stages that lead to senescence following an H_2_0_2_ stress stimulus to proliferating MSCs. We collected cell samples at several time points (0.5 h, 1 h, 24 h, 48 h, and 64 h) following stress (Figure 3A). These points were indicated as SEN1, SEN2, SEN3, SEN4, and SEN5 (Figure 3B–G). We detected a strong increase in the percentage of cells expressing SA-β-gal (54.0%) soon after stress (SEN1), and this percentage declined (30.0%) at SEN2 and then gradually increased to reach its maximum value (63.0%) at SEN5 (Supplemental File S2). It is well-known that, following a genotoxic injury, cells may enter senescence through a multistep process that requires days to reach full accomplishment. In this context, the rapid increase in SA-β-gal activity is not indicative of a senescence status; rather, it may indicate stressed cells that either successfully recover from stress or, alternatively, succumb to the noxious stimulus and enter senescence. We attempted to discriminate between these different outcomes by evaluating the expression of Ki67 and pRPS6 in SA-β-gal positive cells. In this way, we identified three phenotypes: Ki67 (+) pRPS6 (+) SA-β-gal (+) cells we referred to as stressed cells; Ki67 (+) pRPS6 (−) SA-β-gal (+) and Ki67 (−) pRPS6 (−) SA-β-gal (+) we indicated as being in pre-senescence, and Ki67 (−) pRPS6 (+) SA-β-gal (+) cells that represented the senescence (Table 2).

This classification considers that, following stress, cycling cells arrest cell-cycle progression, either in G1 or G2/M, to repair damage induced by a noxious stimulus; however, they do not permanently exit the cell cycle [21,22]. In contrast, irreparable damage may trigger a definitive cell-cycle exit and senescence, which may proceed initially through the acquisition of a generic G0 state with silenced/reduced protein synthesis and, subsequently, with the implementation of senescence-specific programs, which require active protein synthesis [5]. According to the proposed classification, we observed that the percentage of stressed cells strongly increased soon after H_2_0_2_ treatment (time SEN1) and then declined almost to basal value at SEN5 (Figure 3D). Pre-senescent and senescent cells showed increases at late time points (SEN4 and SEN5) (Figure 3E–G). It should be underlined that we did not observe a previous increase in the percentage of pre-senescent cells and then of senescent cells. This may indicate that senescence is a highly heterogeneous phenomenon and that cells may become fully senescent according to different time frames. This heterogeneity may also come from the asynchronous cell state when we treated MSC cultures with H_2_O_2_.

**Figure 4:**
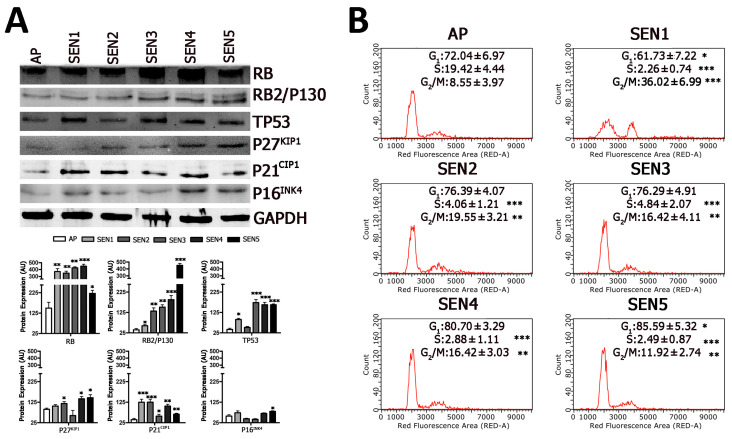
Cell cycle and Western blot analysis of senescent cells. Panels (**A**,**B**): Cell-cycle profiles and western blot analysis of MSC samples collected at different time points following hydrogen peroxide treatment. The * indicates the statistical difference between AP, chosen as the reference, and the other time points (* *p* < 0.05, ** *p* < 0.01, *** *p* < 0.001). In panel (**A**), the graphs show the quantification of western blot bands that was performed by using GAPDH as the loading control.

In a previous finding, we provided evidence that, in MSCs, the RB1 protein plays a role in cell-cycle exit and early senescence but is clearly dispensable for late senescence. This latter stage is supported by RB2/P130 [23]. In the current study, the time-course analysis of RB1, RB2/P130, and the other regulators of cell cycle and senescence clearly showed that, following H_2_0_2_ injury, full-senescence status was reached at later time points and not soon after the increase of SA-β-gal activity (Figure 4A). Indeed, we detected a progressive increase of RB2/P130 and its related CKI (P27^KIP1^), while RB increased at SEN1-4 and then declined at SEN5.

The cell-cycle analysis of cells treated with H_2_0_2_ evidenced a decline of cells in S-phase soon after damage (time SEN1). This percentage increased at SEN2 and SEN3 and then declined again (Figure 4B), and this bimodal pattern may indicate that, soon after noxious stimulus, damaged cells leave the cell cycle. Then, those that successfully recover from damage re-enter the cell cycle, while cells with irreparable damage become senescent.

It should be underlined that the great majority of MSCs following treatment with H_2_0_2_ entered senescence. Nevertheless, after H_2_0_2_ treatment, some cells died by apoptosis (Supplemental File S4). At each time point after stress, a fraction of cell population become apoptotic and did not contribute to cell cycle profiles. This occurrence renders flow cytometry profiles observed at SEN1-5 not directly correlated with AP flow cytometry analysis.

The unusual cell cycle profiles we detected following H_2_0_2_ treatment, with a very significant increase of G2/M cells soon after stress event (SEN1), induced us to evaluate the effect of H_2_0_2_ stress on other cell lines, such as human fibroblasts (Supplemental File S5). In human fibroblasts, we observed a progressive increase of SA-β-gal positive cells after stress induction. This event was associated with a reduction of cells in S-phase and an increase of those in G2/M at SEN4 (Supplemental File S5). These data may suggest that MSCs have a peculiar behavior following genotoxic stress. Indeed, also in a previous investigation with other stressors (X-rays or alpha particles), we observed an increase in G2/M soon after treatments [24].

## 3. Discussion

Quiescence, senescence, and differentiation are different forms that non-cycling cells may take. A clear-cut characterization of the G0 state that is associated with these non-proliferative conditions is quite difficult. These conditions are progressive phenomena, and there is no unique event that represents a turning point indicating that a cell has left the cell cycle and entered quiescence. Instead, it is a step-by-step event due to the fact that the cell progressively rather than abruptly modifies gene expression, metabolism, and functions in order to acquire this new phenotype. The same process occurs for senescence and differentiation. We combined several well-known cell-cycle and senescence markers to identify different steps that lead to full quiescence or senescence. For this latter phenotype, we attempted to discriminate between stress stimuli with which cells successfully cope and those that trigger senescence.

In our experimental model, healthy cells (SA-β-gal negative) express both Ki67 and pRPS6 when in a cycling state. This condition is due to active protein synthesis occurring in proliferating cells and to the Ki67 contribution to normal cell-cycle progression through different functions, such as the formation of a mitotic perichromosomal layer [9,25,26]. Quiescent cells (G0) do not express either Ki67 or pRPS6, and this state is due to minimal protein synthesis during quiescence and the dispensability of Ki67 for maintaining quiescence [7,9]. The progression from the cycling state to G0 is a multistep process, and before acquisition of the full quiescent condition, cells leaving the cell cycle may pass through several G0-entry states [27,28]. We hypothesized that healthy cells, which still express Ki67 but are negative for pRPS6, may represent a cell population in a G0-entry condition. Indeed, a prerequisite for acquiring the quiescent phenotype is to adjust energy demand by reducing protein synthesis. At the same time, proteins produced before slowing down the protein translation may still be present in G0-entry cells. In accordance with this hypothesis, there are findings claiming that residual Ki67 expression can be detected in G0 cells [10]. This residual expression may be present in a cell subpopulation representing the G0-entry cells; indeed, cells leaving the cell cycle are not a homogeneous population.

SA-β-gal is a lysosomal enzyme that has been widely used as a senescence marker, but its inactivation did not reverse the onset of senescence. Nevertheless, the alteration of lysosomal functions is strictly related to senescence and aging [29,30]. Lysosomes can be considered sensors that contribute to the adaptation of cellular metabolism and functioning to both intrinsic and extrinsic stimuli. Several cellular stressors trigger lysosome activation to remove damaged macromolecules and organelles [31]. Stress, however, may alter lysosome function by modifying the pH of its lumen. Lysosomes have an acidic lumen in which dozens of hydrolytic enzymes are activated; stress can increase the pH of the lumen, thus inhibiting the activity of acidic hydrolases and, potentially, promoting the activities of other neutral (or quasi-neutral) hydrolases [32,33]. In this context, it is reasonable to hypothesize that, following stress, SA-β-gal, which works at pH 6.0, is activated, and then its activity declines if lysosomes successfully cope with the stress stimulus. Alternatively, permanent cellular damage induced by stress may be associated with continuous SA-β-gal activity. Our results lend credence to this hypothesis. Soon after reactive oxygen stress (0.5 h), cells expressed high levels of SA-β-gal activity. These cells were also positive for Ki67 and pRPS6 and, hence, were not senescent cells. Senescence onset, indeed, requires several steps and cannot occur in minutes following stress. In our experiments, we detected a sudden increase in SA-β-gal activity followed by a decline (1 h) and, hence, a further progressive increase (24, 48, and 64 h post-stress). This second wave of SA-β-gal activity may be considered associated with the onset of senescence. Indeed, this activity was associated with Ki67(+) pRPS 6 (−), Ki67(−) pRPS6(−), and Ki67 (−) pRPS6 (+) phenotypes; the first two phenotypes may represent pre-senescent stages, and the last one may be associated with senescence. A cell must definitively leave the cell cycle to become senescent. This process may start with the silencing of Ki67 activity and protein synthesis (early senescence), after which the cell reactivates protein synthesis to accomplish the new activities related to its senescent status (late senescence).

The previous studies of our group further support the hypothesis that the bimodal increase in SA-β-gal activity may be related to temporary and permanent stress conditions. Following genotoxic stress, cells showed DNA injury. DNA damage triggers the activation of ATM that promotes H2AX phosphorylation (γ-H2AX). The γ-H2AX foci in the nuclei indicate damaged DNA that is being repaired. Soon after stress events, the presence of γ-H2AX foci pinpoint active repair, while foci persistence several hours or days following stress indicates the presence of unrepaired or DNA. In MSCs treated with hydrogen peroxide or X-ray, we did a follow-up of γ-H2AX foci. The γ-H2AX foci increased soon after stress and then declined, but some cells retained H2AX foci [24,34]. These were senescent cells that displayed unrepaired DNA, while those that successfully repaired DNA within hours of a noxious stimulus may be considered stressed cells that successfully coped with the burden. It remains to evaluate whether cells that express SA-β-gal activity soon after a stress event and present γ-H2AX foci may progressively become both SA-β-gal and γ-H2AX negative cells.

## 4. Conclusions

The identification of different G0 states is fundamental in order to better evaluate quiescence, senescence, and differentiation. We provided a simple method that allows this identification within a cell population of quiescent and senescent phenotypes. We also provided evidence that SA-β-gal activity can be considered to be associated with senescence only after several hours following a genotoxic event. A definitive senescent phenotype can be identified by evaluating the expression of Ki67, pRPS6, and SA-β-gal activity.

A limit for our study and for similar findings relies on the consideration that the entering in quiescence or in senescence is continuous phenomenon and hence discrete conditions do not exist. The classification we adopt refers to “key turning points” of transitional states that continuously change.

## 5. Material and Methods

### 5.1. Cell Cultures

The experimental procedures followed the rules approved by the Ethics Committee of the Luigi Vanvitelli Campania University. Patients were informed of the research and gave permission for the use of biological samples. Bone marrow was obtained from three healthy donors. Cells were separated through Ficoll density gradient (GE Healthcare, Chicago, IL, USA), and the mononuclear cell fraction was collected and washed in phosphate-buffered saline solution (PBS, Microgem, Naples, Italy). We seeded 1 to 2.5 × 10^5^cells/cm^2^ in alpha-minimum essential medium (alpha-MEM, Microgem) supplemented with 10% fetal bovine serum (FBS, Euroclone, Pero, Italy) and 1 ng/mL beta-fibroblast growth factor (β-FGF, Prepotech, London, UK). After 72 h, non-adherent cells were discarded, and adherent cells were further cultivated to reach confluency. We verified that, under our experimental conditions, the bone-marrow stromal cultures contained MSCs that fulfilled the three criteria proposed to define MSCs [35]. All experiments were conducted on MSC cultures at passage 3 when senescence phenomena were minimal [36].

### 5.2. Induction of Quiescence and Cell-Cycle Re-Entry

MSC cultures at passage 3 were incubated in alpha-MEM supplemented with 0.1% FBS and 1 ng/mL β-FGF. Cells were cultivated for 36 h in these conditions; then the medium was replaced with a new one containing 10% FBS. The cells were grown in this new medium for another 36 h. At several time points before and after the medium change, cell samples were collected for other biological assays.

### 5.3. Induction of Acute Senescence

MSC cultures at passage 3 (70% confluent were incubated with 300 μM hydrogen peroxide (H_2_O_2_) (Sigma-Aldrich, St. Louis, MO, USA) for 0.5 h. Following this treatment, cells were further cultivated for 64 h. Cell samples were collected at 0.5 h, 1 h, 24 h, 48 h, and 64 h post-H_2_O_2_ treatment for other biological assays.

### 5.4. Cell-Cycle Analysis

For each analysis, 5 × 10^4^ cells were collected by trypsin treatment and then, after PBS washing, were fixed in 70% ethanol overnight at −20 °C. The samples were then washed with PBS and subsequently dissolved in a hypotonic buffer containing propidium iodide (Muse TM Cell Cycle Kit cat. MCH100106, Millipore, Burlington, MA, USA). Analyses were performed using Muse Cell Analyzer (Millipore, MA, USA) following the manufacturer’s instructions.

### 5.5. Immunocytochemistry and Senescence-Associated Beta-Galactosidase

For the beta-galactosidase assay, 20,000 cells per well were seeded in 24 wells with glass coverslips. After several experiments were conducted, cells were fixed in a solution of 2% formaldehyde for 10 min. Then, cells were washed with PBS (Microgem) and incubated at 37 °C overnight with a staining solution (citric acid/phosphate buffer (pH 6), K4Fe (CN) 6, K3Fe (CN) 6, NaCl, MgCl2, X-Gal). Cells were then permeabilized with 0.3% Triton-X100 (Roche, Basilea, Switzerland) on ice for 5 min and then incubated in a blocking solution (5% FBS solution in PBS and 0.1% Triton-X100) for 1 h at room temperature (RT). Subsequently, samples were incubated with the antibodies against pRPS6 (1:1000, 4858, Cell Signaling, Danvers, MA, USA) and Ki67 (1:200, sc7846, SantaCruz Biotech, Santa Cruz, CA, USA) at 4 °C overnight. We then used FITC-conjugated secondary antibody, goat anti-rabbit (1:400, Gtx-Rb-003D488), or TRITC-conjugated secondary antibody goat anti-mouse (1:400, Gtx-Mu-003D594), which were obtained from ImmunoReagents (Raleigh, NC, USA). Nuclear staining was performed using DAPI mounting medium (ab104139, ABCAM, Cambridge, UK), and micrographs were performed with a fluorescence microscope (Leica DM2000, -DMC5400, Leica, Wetzlar, Germany). For every marker we analyzed, the percentage of positive cells was calculated by the number of cells that expressed the specific marker stain out of at least 500 cells in different microscope fields.

### 5.6. Western Blot

Cells were lysed in a buffer containing 0.1% Triton (Bio-Rad, Hercules, CA, USA) for 30 min in ice; 20 μg of each lysate was electrophoresed in a polyacrylamide gel and electroblotted onto a nitrocellulose membrane. We used the following primary antibodies: RB1 (AV33212) and GAPDH (G8795) from Sigma-Aldrich (St. Louis, MO, USA); RB2/P130 (R27020) from BD Biosciences (Allschwil, Switzerland); and P27^KIP1^ (3686) from Cell Signaling Technology (Danvers, MA, USA), while TP53 (sc-126) and P21^CIP1^ (sc-397) were obtained from Santa Cruz Biotechnology (Santa Cruz, CA, USA), and P^16INK4A^ (ab54210) was obtained from Abcam (Cambridge, UK). Immunoreactive signals were detected with a horseradish peroxidase conjugated secondary antibody (ImmunoReagents, Raleigh, NC, USA) and reacted with ECL plus reagent (Merck Millipore, Burlington MA, USA). All antibodies were used according to manufacturer’s instructions. The mean value was quantified densitometrically using Quantity One^®^ 1-D analysis software (Bio-Rad, Hercules, CA, USA).

### 5.7. Statistical Analysis

Statistical significance was evaluated using ANOVA analysis followed by Student’s t and Bonferroni’s tests. A mixed-model variance analysis was used for data with continuous outcomes. All data were analyzed with GraphPad Prism version 5.01 statistical software package (GraphPad, San Diego, CA, USA).

## Figures and Tables

**Table 1:** Molecular algorithm to identify cell phenotypes.

Phenotype	Ki67	pRPS6	SA-β-Gal
cycling cells	+	+	−
G0-entry	+	−	−
G0	−	−	−
G0-alert	−	+	−

**Table 2:** Molecular algorithm for identifying cell phenotypes.

Phenotype	Ki67	pRPS6	SA-β-Gal
stressed cells	+	+	+
pre-senescence	+	−	+
	−	−	+
senescence	−	+	+

## Data Availability

Further information regarding data contained in the article may be provided upon request.

## Supplementary Materials

The following are available online at https://www.mdpi.com/1422-0067/22/6/3102/s1. Supplemental File S1. Molecular characterization of cycling and quiescent cells. The panels show further representative micrographs of MSCs stained to identify nuclei (DAPI), pRPS6, and Ki67 and to evaluate SA-β-gal activity. The terms: AP, S1, S2, S3, Q, T1, T2, T3, P indicate different time points. Supplemental File S2. SA-β-gal staining of MSC cultures. The panels show f representative micrographs of MSCs staining to evaluate SA-β-gal activity (blue). The terms: AP, SEN1, SEN2, SEN3, SEN4, SEN5 indicate different time points. Supplemental File S3. Molecular characterization of senescent cells. The panels show further representative micrographs of MSCs stained to identify nuclei (DAPI), pRPS6, and Ki67 and to evaluate SA-β-gal activity. The terms: AP, SEN1, SEN2, SEN3, SEN4, SEN5 indicate different time points. Supplemental File S4. Apoptosis detection by Annexin-V Assay. The picture shows representative FACS analysis of MSC apoptosis. The assay identifies early (Annexin V + and 7ADD −) and late apoptosis (Annexin V + and 7ADD +). Apoptosis is a continuous process, and we calculated the percentage of apoptosis as the sum of early and late apoptotic cells. The histogram shows the mean percentage of Annexin V-positive cells. The Guava EasyCyte (Merck Millipore, Italy) flow cytometer was used to evaluate apoptotic cells using a kit of fluorescein conjugated with Annexin V, following the manufacturer’s instructions. Apoptotic and non-apoptotic cells were analyzed and identified by two separate dyes (Annexin V and 7AAD) to cover a broad spectrum of cells: phosphatidylserine is bound by Annexin V (green) on the external membrane of apoptotic cells, while 7AAD (red) permeates and stains DNA in late-stage apoptotic and dead cells. Coloration enables the identification of 3 cell populations: non-apoptotic cells (Annexin V− and 7AAD+); early apoptotic cells (annexin V+ and 7AAD-); and late apoptotic or dead cells (Annexin V + and 7AAD +). In our experimental conditions, both the early apoptotic and late apoptotic cells were grouped. Supplemental File S5. Treatment of human dermal fibroblasts with H_2_O_2_. Human BJ-5ta dermal fibroblasts (HDF) from ATCC Italy (code CRL-4001) were grown in DMEM low glucose with 10% FBS. HDF cultures (70% confluent) were incubated with 300μM hydrogen peroxide (H_2_O_2_) (Sigma-Aldrich MO, USA) for 0.5 h. Following this treatment, cells were further cultivated for 64 h. Cell samples were collected at 0.5 h, 1 h, 24 h, 48 h, and 64 h post-H_2_O_2_ treatment for other biological assays. The term AP stands for active proliferating cells. The terms SENs refer to incubation time after H_2_O_2_ treatment: SEN1 (0.5 h), SEN2 (1 h), SEN3 (24 h), SEN4 (48 h), SEN5 (64 h). In the table are reported the percentage of senescent cells detected by determining SA-β-gal activity. The cell cycle was evaluated with as reported in Methods. The apoptosis was detected as described in Supplemental File S4.
