# Supplementary material for: Different Stages of Quiescence, Senescence, and Cell Stress Identified by Molecular Algorithm Based on the Expression of Ki67, RPS6, and Beta-Galactosidase Activity
Source: Int J Mol Sci. 2021 Mar 18;22(6):3102. doi: 10.3390/ijms22063102 (PMC8002939; doi:10.3390/ijms22063102)
Supplement: Supplementary file 1 [file ijms-22-03102-s001.zip › Suppl File 5.pdf]

	AP	SEN1	SEN2	SEN3	SEN4	SEN5
**Senescent cells**	6.8±0.9	27.1±3.5	18.5±2.4	15.5±2.32	20.1±2.4	28.2±3.6
**G0/G1**	70.7±12.1	81.3±8.9	78.3±13.3	82.1±13.9	54.8±7.1	55.5±6.6
**S**	14.41±0.4	8.4±0.5	6.5±0.8	4.8±0.7	6.0±0.9	5.57±0.8
**G2/M**	14.8±2.1	10.2±1.8	15.0±1.8	13.0±1.8	39.1±6.6	38.8±6.6
**Apoptotic cells**	3.0±0.4	5.6±0.7	2.3±0.5	6.7±0.8	5.2±0.3	7.1±0.7

Supplemental file 5 – Treatment of human dermal fibroblasts with H_2O_2

Human BJ-5ta dermal fibroblasts (HDF) from ATCC Italy (code CRL-4001) were grown in DMEM low glucose with 10% FBS. HDF cultures (70% confluent) were incubated with 300μM hydrogen peroxide (H_2O_2) (Sigma-Aldrich MO, USA) for 0.5 hour. Following this treatment, cells were further cultivated for 64 hours. Cell samples were collected at 0.5, 1, 24, 48, and 64 hours post-H_2O_2 treatment for other biological assays.

The term AP stands for active proliferating cells. The terms SENs refer to incubation time after H_2O_2 treatment: SEN1 (0.5 hours), SEN2 (1 hour), SEN3 (24 hours), SEN4 (48 hours), SEN5 (64 hours).

In the table are reported the percentage of senescent cells detected by determining SA-β-gal activity. The cell cycle was evaluated with as reported in Methods. The apoptosis was detected as described in supplemental file 4.
