# Supplementary figures and images for: Different Stages of Quiescence, Senescence, and Cell Stress Identified by Molecular Algorithm Based on the Expression of Ki67, RPS6, and Beta-Galactosidase Activity
Source: Int J Mol Sci. 2021 Mar 18;22(6):3102. doi: 10.3390/ijms22063102 (PMC8002939; doi:10.3390/ijms22063102)

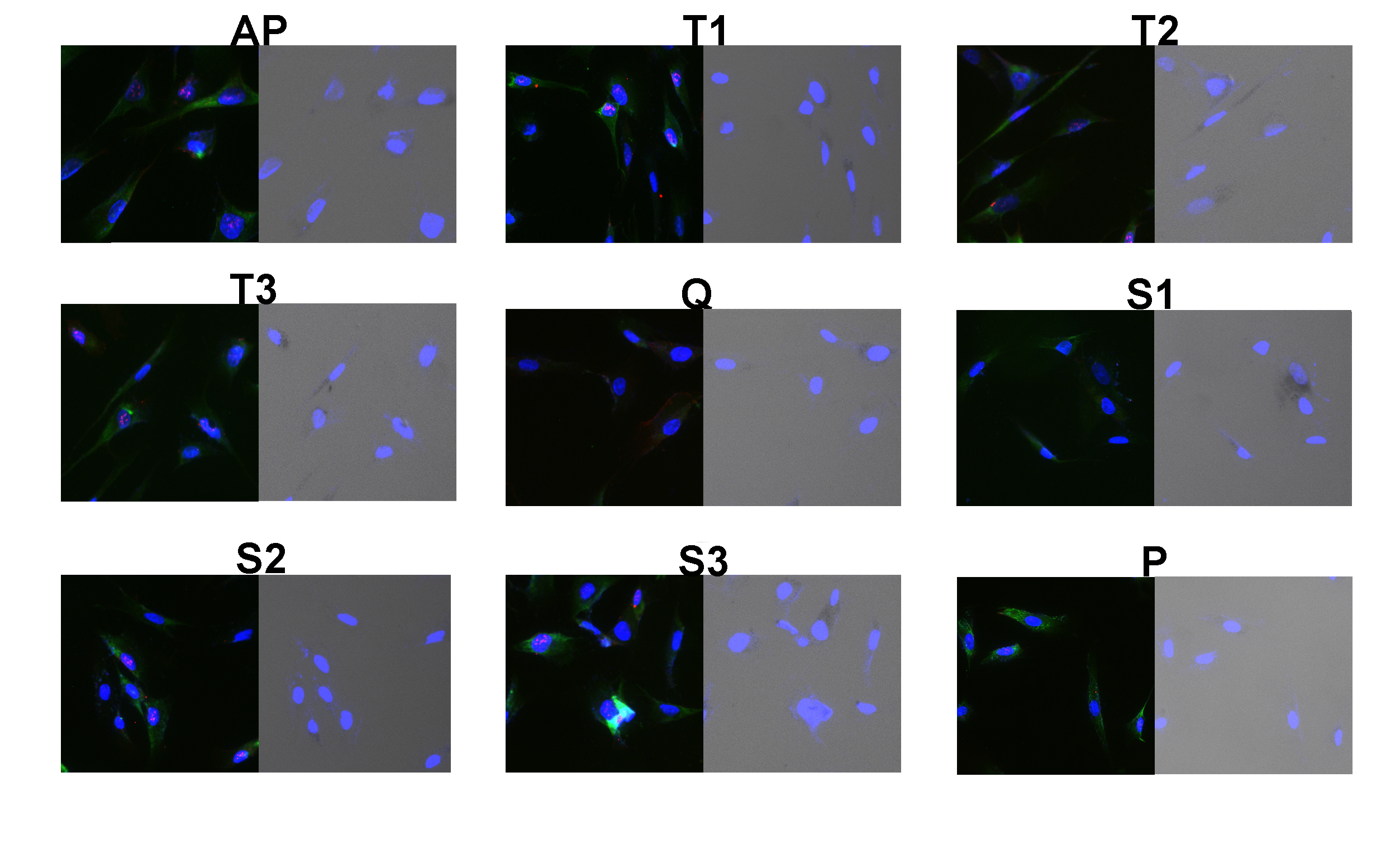

Supplement: Supplementary file 1 [file ijms-22-03102-s001.zip › Supp File 1.tif]

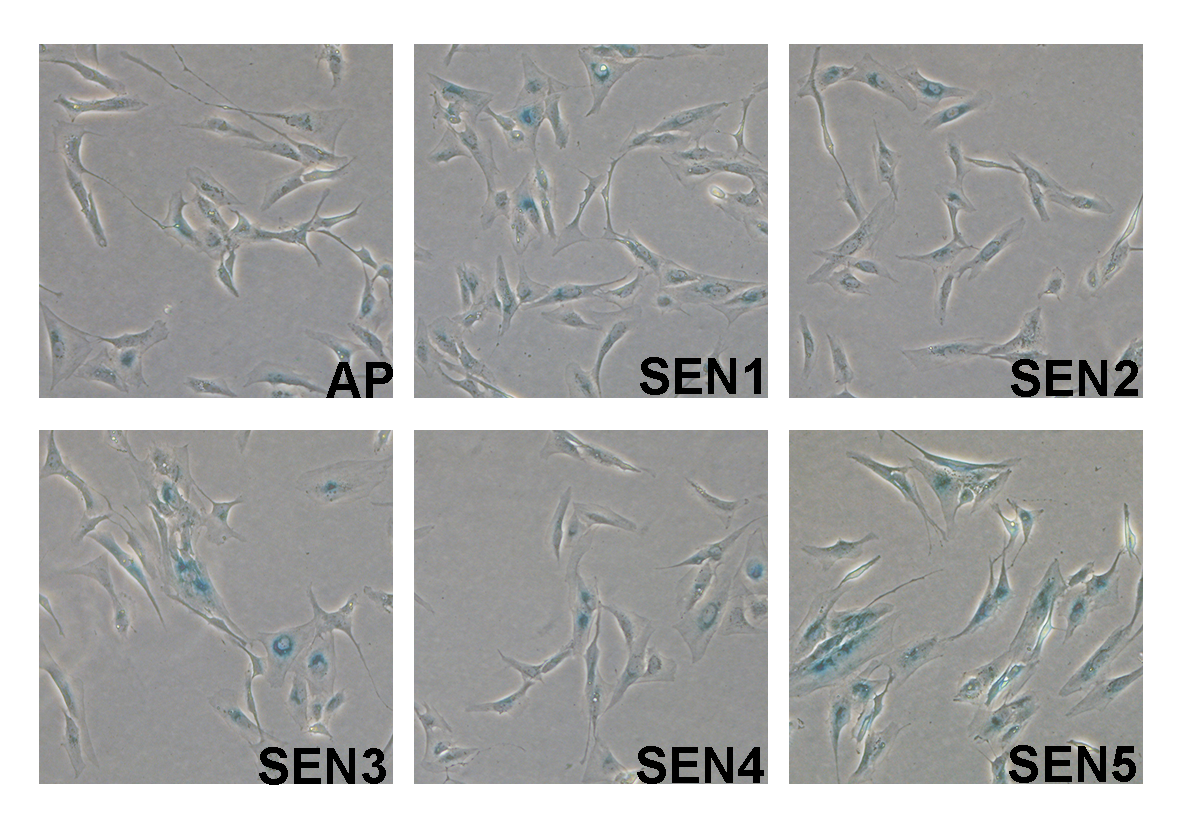

Supplement: Supplementary file 1 [file ijms-22-03102-s001.zip › Supp File 2.tif]

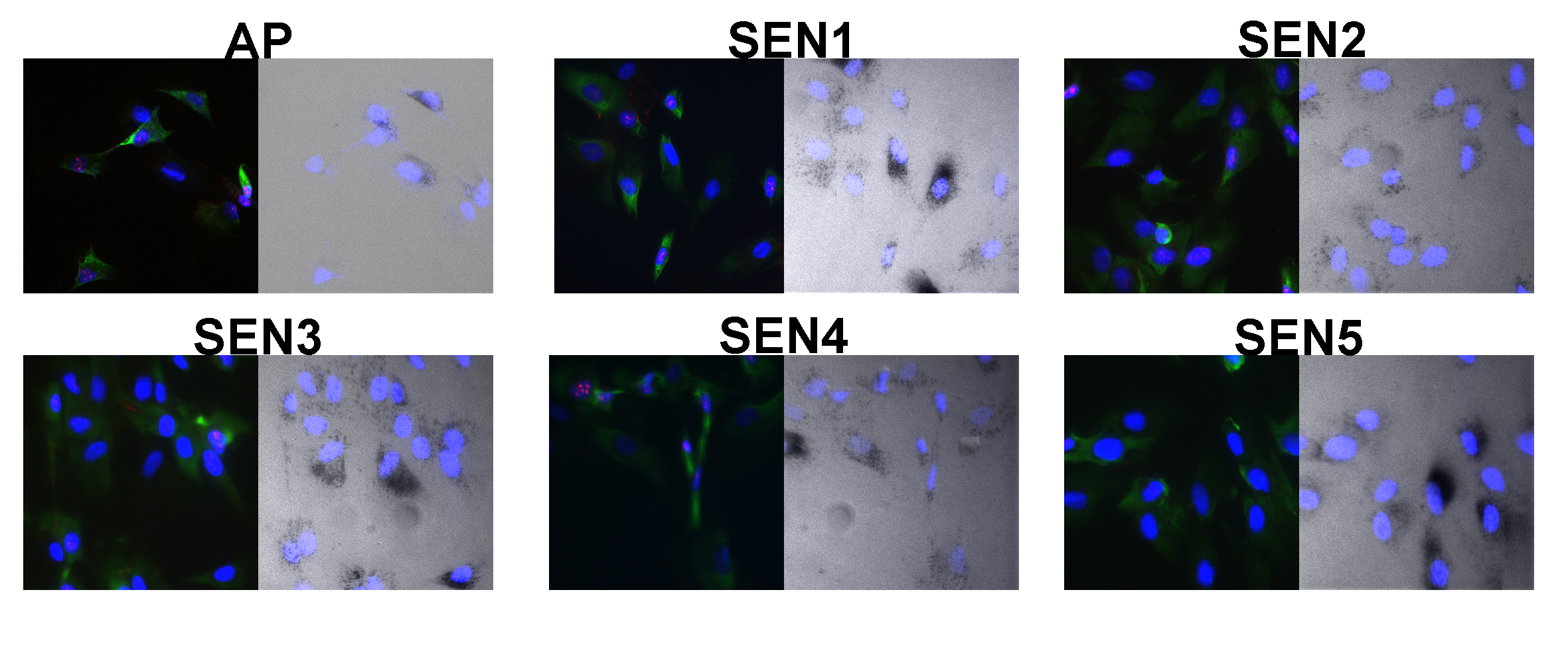

Supplement: Supplementary file 1 [file ijms-22-03102-s001.zip › Supp File 3.tif]

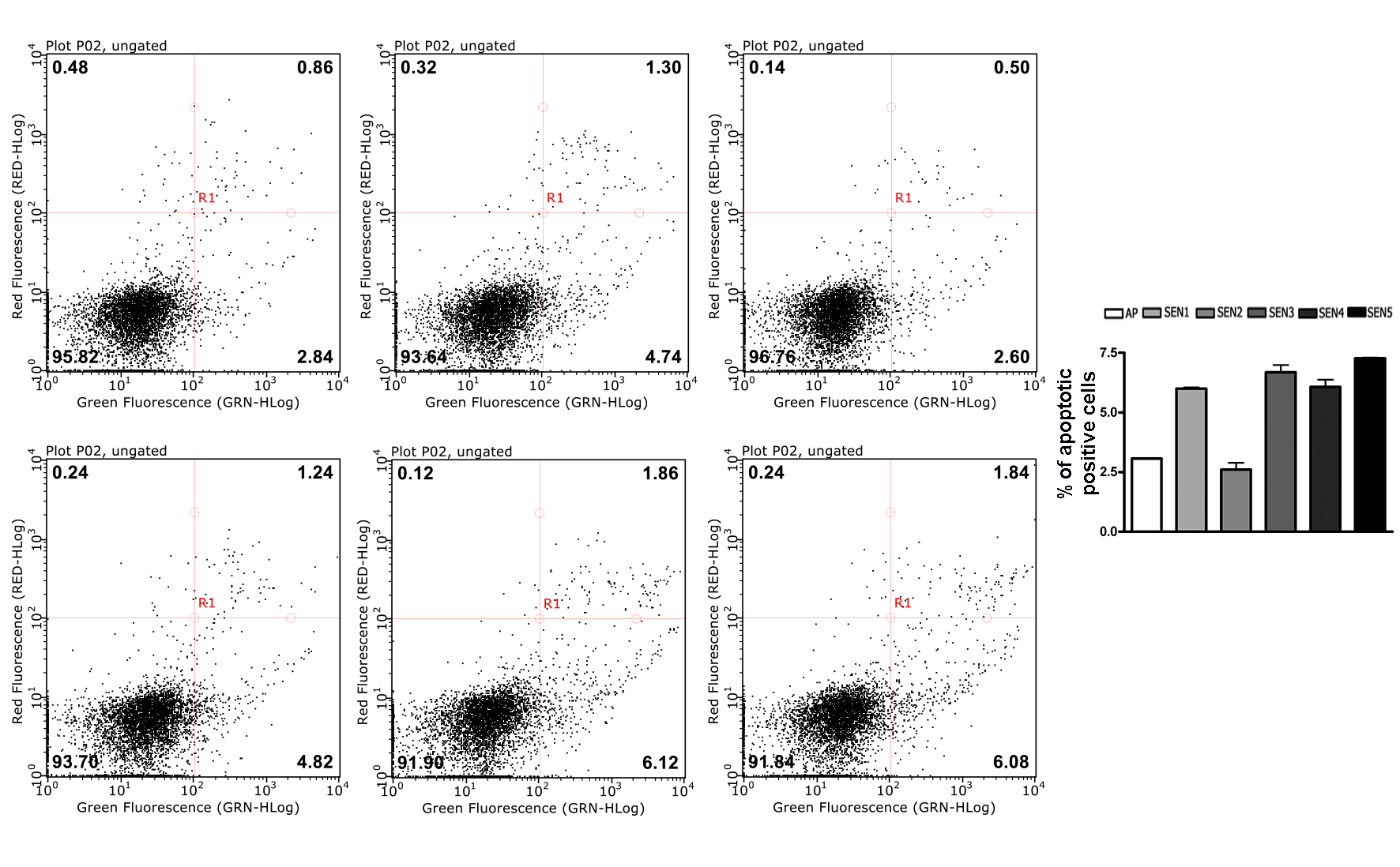

Supplement: Supplementary file 1 [file ijms-22-03102-s001.zip › Supp File 4.tif]
